# An updated profile of the cancer burden, patterns and trends in Latin America and the Caribbean

**DOI:** 10.1016/j.lana.2022.100294

**Published:** 2022-09

**Authors:** Marion Piñeros, Mathieu Laversanne, Enrique Barrios, Marianna de Camargo Cancela, Esther de Vries, Constanza Pardo, Freddie Bray

**Affiliations:** a Cancer Surveillance Branch, International Agency for Research on Cancer, Lyon, France; b National Cancer Registry, Honorary Commission for the Fight against Cancer, Montevideo, Uruguay; c Cancer Surveillance Division, National Cancer Institute INCA, Rio de Janeiro, Brasil; d Department of Clinical Epidemiology and Biostatistics, Pontificia Universidad Javeriana, Bogotá, Colombia; e Cancer Surveillance Group, National Cancer Institute, INC Bogotá, Colombia

**Keywords:** Neoplasms, Cancer, Global health, Epidemiology, Public health surveillance

## Abstract

**Background:**

Cancer is a leading cause of disease and death in Latin America and the Caribbean (LAC). Contemporary data on the cancer burden aims to inform effective cancer policies; this article provides an update and benchmarking of national cancer incidence and mortality estimates for the year 2020, alongside recent mortality trends in the region.

**Methods:**

The number of new cancer cases and deaths were extracted from the GLOBOCAN 2020 database developed by the International Agency for Research on Cancer (IARC), and mortality data over time from IARC's cancer mortality database, New cancer cases, deaths and corresponding age-standardized rates per 100,000 person-years are presented. Random fluctuations in mortality trends by country, sex and cancer site were smoothed using LOWESS regression.

**Findings:**

An estimated total of 1.5 million new cancer cases and 700,000 deaths occur annually in LAC, with corresponding incidence and mortality rates of 186.5 and 86.6 per 100,000. The most common cancers in 2020 were prostate (15%), breast (14%), colorectal (9%), lung (7%) and stomach (5%). Lung cancer remained the leading cause of cancer death (12%), though rates varied substantially between countries. The mortality trends of infectious-related cancers tended to decline in most countries, while rates of cancer types linked to westernization were mainly increasing. Assuming rates remain unchanged, the cancer burden in LAC will increase by 67% reaching 2.4 million new cases annually by 2040.

**Interpretation:**

The cancer patterns reflect important underlying sociodemographic changes occurring over the last decades. With an increasing burden anticipated over the next decades in this region, there is a need to plan oncological service provision accordingly.

**Funding:**

No external funds received.

**Research in context**

**Evidence before this study**

Comparative studies on the cancer patterns and trends in the regions of South and Central America and the Caribbean have been published previously. In recent years, the number and scope of descriptive studies of cancer have also increased at the country level. There is however a need for a comprehensive description of the current situation across LAC, based on the most recent GLOBOCAN estimates.

**Added value of this study**

We provide an updated overview of estimates for cancer incidence and mortality in the year 2020 for 32 countries in a region that is undergoing important sociodemographic change. Updated mortality trends for the main cancers provide valuable information on impact. The findings are discussed in light of current available regional and local evidence on the underlying determinants and the prospects of effective cancer control. The study also fosters international collaborative research with local partners in the LAC region that are actively supporting the Global Initiative for Cancer Registry Development (GICR, http://gicr.iarc.fr).

**Implications of all the available evidence**

This study stimulates and facilitates comparisons with observed data from subnational population-based cancer registries, which, together with vital statistics, constitute the foundation for IARC's global estimates. Such an exposition of current patterns, recent trends and future projections, provides an evidence base for local actors seeking to develop cancer control interventions in their respective countries, as well as a baseline reference for further studies in the region. Despite the existence of estimates, countries need to do an effort to improve the generation of data by population-based cancer registries.

Alt-text: Unlabelled box

## Introduction

The Latin America and the Caribbean (LAC) region has doubled in population size over the last half century to 685 million inhabitants by 2020. Concurrently, life expectancy has increased among the 32 constituent countries reaching 76 years at the regional level, though national averages vary markedly – from 65 to 83 years (in Guyana and Martinique, respectively).^1^ With cancer already being the leading cause of premature death in almost half of the LAC countries,^2^ and the cancer burden predicted to rise over the next decades,^3^ there are major challenges ahead in planning rational cancer care and preventive services in a region where one in three are living in poverty.^1^

In this report, we examine national and regional cancer incidence and mortality patterns using the most recent GLOBOCAN estimates for the year 2020, alongside recent national mortality trends using the WHO mortality database.^4^ To enable benchmarking in the region and to facilitate comparisons with our previous report^5^ we additionally provide comparisons with corresponding estimates in the U.S. and Spain for the same year using the same sources.

## Methods

The number of new cancer cases and deaths were extracted from the GLOBOCAN 2020 database for the LAC countries (in addition to the U.S. and Spain), by sex and 18 age groups (0-4, 5-9, ..., 80-84, 85 and over).^6^ Corresponding population data for 2020 were extracted from the United Nations (UN) website. The data sources and hierarchy of methods used in compiling the cancer estimates have been described in detail elsewhere.^7^ In brief, the GLOBOCAN estimates are assembled at the national level using the best available sources of cancer incidence and mortality data within a given country. These can be high-quality cancer registry incidence data as compiled in the Cancer Incidence in Five Continents Series,^8^ new data sources most notably in sub-Saharan Africa, targeted searches for new registry data online and the most recent mortality data from the WHO. The methods used to derive the 2020 estimates correspond to those used to derived for previous years^9^, ^10^, ^11^; where applicable, priority is given to short-term predictions and modelled mortality to incidence (M:I) ratios, while validity is dependent on the degree of representativeness and quality of the source information.^7^ As such, the methods of estimation are country-specific, and the quality of the national estimates depends on the coverage, accuracy and timeliness of the recorded incidence and mortality data in a given country. The interested reader can consult the methods used for each individual country in the respective section of the Global Cancer Observatory.^12^ As outlined previously,^13^ the incidence estimates do not take into account the effects of the COVID-19 pandemic and the indirect disruption to cancer services already reported^14^^,^^15^ that may influence the recorded number of new cases of cancer and cancer deaths in future years in the region.

We present tables and figures based on the estimated new cases and deaths, as well as the age-standardized (incidence or mortality) rate (ASR) per 100,000 person-years based on the 1966 Segi-Doll World standard population and using direct standardization. These measures allow comparisons between populations adjusted for differences in age structures.

Incidence trends using the limited high-quality cancer registry data in the region were the subject of a previous paper.^5^ In this report, we assess national progress in cancer control by examining mortality trends using IARC's cancer mortality database containing selected cancer mortality statistics by country, extracted from the WHO database; the mortality data from the U.S. and Spain used for comparative purposes, were from the same source. We examine the six most common forms of cancer death in the region, namely: cancers of the stomach (ICD-10 C16), colon and rectum (C18-21), lung (including trachea, C33-34), female breast (C50), cervix uteri (C53) and prostate (C61). To limit misclassification bias due to high proportion of uterine deaths unspecified to corpus or cervix in some LAC countries, we combined ICD-10 C53 (cervix) and C55 (uterus unspecified) and present trends restricted to ages under 50 years, given the relative rarity of premenopausal endometrial cancer deaths.

To reduce the random fluctuations in the yearly mortality trends, the annual rates were smoothed using lowess regression by country, sex and cancer site. Lastly, we present predictions of the all-cancer incidence by subregion for the year 2040, based on country-, sex-, and age-specific incidence rates and UN population projections, assuming national rates in 2020 remain constant to 2040.

The results are presented by country, and aggregated across the three UN subregions: South America, Central America and the Caribbean. We have also included information on national levels of the UN's Human Development Index (HDI) in 2020,^16^ as well as grouped countries using the four-tier classification (low, medium, high and very high HDI). The Global Cancer Observatory (GCO, https://gco.iarc.fr) includes facilities for the tabulation and graphical visualization of the GLOBOCAN database, including explorations of the current and future burden for 36 cancer types, and all cancers combined, including non-melanoma skin cancer (ICD-10 C44 excluding basal-cell carcinomas).

### Role of the funding source

No external funds received.

## Results

### Overall cancer burden in LAC

For males and females combined, the 2020 estimates indicate that almost 1.5 million new cancer cases and 700,000 deaths occurred annually in the LAC region. New cancer cases in the region accounted for 7.6% of all cases worldwide. The annual number of new cancers in children aged 0-14 years (20,855; data not shown) represented 1.5% of the total new cancer cases. The overall cancer incidence and mortality rates (ASR per 100,000) for LAC were 186.5 and 86.6 respectively (Table 1). Comparing the three subregions, South America displayed the highest incidence rates for all cancers and both sexes combined; highest country-level incidence (ASR per 100.000) rates were seen in Uruguay (269.3 in both sexes) and Martinique (248.7) more than doubling the (lowest) rates from Guatemala (123.1) and Belize (120.9). In terms of cancer mortality, Uruguay and Barbados exhibited the highest rates, while Belize and Mexico the lowest (Table 1). In general, the Central American countries displayed the lowest incidence and mortality rates. The M:I ratios, which can be interpreted as a surrogate of case-fatality, were highest in the Caribbean, with Haiti and Barbados having the highest ratios: 0.75 and 0.65, respectively. In most countries, all-cancer incidence rates were higher among males than females, except for Bolivia, Ecuador, Guyana, Peru, Belize, El Salvador, and Mexico (Table 1). Similarly, mortality rates were consistently higher among males, except for Bolivia, Guyana and Peru.

**Table 1:** Estimated population, human development index and number of cancer cases, deaths and age-standardized incidence and mortality rates (per 100,000) in the LAC countries, 2020.

Region, subregion, and country	Population	Human Development Index		Incidence						Mortality						
				Male		Female		Both sexes		Male		Female		Both sexes		
		Value	Label	Cases	ASR	Cases	ASR	Cases	ASR	Deaths	ASR	Deaths	ASR	Deaths	ASR	M:I ratio
Latin America and the Caribbean	653962327			720267	199.2	750007	178.84	1470274	186.5	365135	98.1	348279	78.3	713414	86.6	0.49
South America				539931	217.1	555417	192.1	1095348	201.4	267301	104.9	254088	82.1	521389	91.5	0.48
Argentina	45195777	0.83	Very high	62327	230.7	68551	213.28	130878	218.2	35742	126.1	34332	92.9	70074	106.1	0.54
Bolivia	11673029	0.703	High	6773	121.4	9044	154.87	15817	137.5	4492	77.2	5443	87.7	9935	82.2	0.63
Brazil	212559410	0.761	High	300114	241.3	292098	198.16	592212	215.4	137259	108.4	122690	78.6	259949	91.2	0.44
Chile	19116209	0.847	Very high	28779	209.2	25448	161.22	54227	180.9	15047	103.8	13537	76.0	28584	87.4	0.53
Colombia	50882884	0.761	High	52866	184.7	60355	182.58	113221	182.3	26862	91.1	28125	80.1	54987	84.7	0.49
Ecuador	17643060	0.758	High	13190	146.3	16083	164.37	29273	154.6	7296	78.2	7827	75.8	15123	76.4	0.52
French Guyana	298682			315	257.6	249	179.59	564	216.2	135	122.1	84	62.5	219	87.6	0.39
Guyana	786559	0.67	Medium	511	132.9	637	152.92	1148	141.5	249	61.9	316	74.5	565	67.7	0.49
Paraguay	7132530	0.724	High	6500	197.6	6420	187.17	12920	191.0	3546	106.9	3019	86.1	6565	95.5	0.51
Peru	32971846	0.759	High	32680	169.9	37169	185.88	69849	176.3	16430	83.7	18546	88.6	34976	85.5	0.50
Suriname	586634	0.724	High	519	186.0	537	159.26	1056	167.5	356	126.2	302	84.4	658	101.5	0.62
Uruguay	3473727	0.808	Very high	8318	325.6	7439	229.77	15757	269.3	4638	166.1	3939	101.3	8577	127.5	0.54
Venezuela	28435943	0.726	High	27037	184.2	31387	181.92	58424	181.1	15249	103.5	15928	90.0	31177	95.0	0.53
Central America				120657	140.9	140989	141.1	261646	140.2	61544	70.2	64527	63.1	126071	66.0	0.48
Belize	397621	0.72	High	184	117.9	211	122.23	395	120.9	111	71.4	102	60.9	213	66.4	0.54
Costa Rica	5094114	0.794	High	6521	194.1	6618	186	13139	188.7	3189	90.1	2839	71.9	6028	80.1	0.46
El Salvador	6486201	0.667	Medium	4097	126.0	5529	132.75	9626	129.6	2343	68.0	2958	66.0	5301	66.8	0.55
Guatemala	17915567	0.651	Medium	7625	125.7	9061	121.76	16686	123.1	4402	70.7	5207	70.9	9609	70.7	0.58
Honduras	9904608	0.623	Medium	5185	142.9	5443	128.57	10628	133.7	3277	90.2	3101	75.1	6378	81.3	0.60
Mexico	128932753	0.767	High	89536	139.7	105963	142.44	195499	140.4	44140	67.2	46082	60.4	90222	63.2	0.46
Nicaragua	6624554	0.651	Medium	3591	141.4	4401	133.6	7992	135.7	2110	82.7	2450	75.3	4560	78.0	0.57
Panama	4314768	0.795	High	3918	159.8	3763	145.32	7681	151.0	1972	76.8	1788	65.1	3760	70.2	0.49
The Caribbean				59679	213.8	53601	174.4	113280	191.7	36290	120.5	29664	89.0	65954	102.7	0.58
Bahamas	393248	0.805	Very high	433	209.2	443	175.03	876	188.9	227	110.1	240	93.5	467	99.8	0.53
Barbados	287371	0.813	Very high	573	228.2	578	214.08	1151	219.2	373	128.6	370	118.1	743	121.9	0.65
Cuba	11326616	0.778	High	25004	239.5	21790	199.29	46794	217.1	15857	139.2	11332	91.0	27189	113.0	0.58
Dominican Republic	10847904	0.745	High	10332	188.7	9484	159.06	19816	172.4	6383	109.2	5724	92.4	12107	99.6	0.61
France, Guadeloupe	400127			1329	349.1	798	184.56	2127	258.6	464	106.4	381	69.7	845	85.4	0.40
Haiti	11402533	0.503	Low	6115	152.9	6289	127.44	12404	137.9	4608	117.9	4637	95.5	9245	104.0	0.75
Jamaica	2961161	0.726	High	3651	206.8	3546	191.51	7197	198.8	2457	129.8	2119	110.6	4576	119.9	0.64
France, Martinique	375265			1244	310.6	912	197.77	2156	248.7	467	96.0	414	67.2	881	79.4	0.41
Puerto Rico	2860840			7001	256.8	6079	192.44	13080	218.6	3116	94.8	2454	59.1	5570	73.9	0.43
Saint Lucia	183629	0.745	High	265	208.0	184	143.04	449	174.3	141	99.9	91	66.5	232	82.8	0.52
Trinidad and Tobago	1399491	0.799	High	1991	207.1	1928	186.11	3919	193.9	1195	119.4	1044	93.3	2239	103.4	0.57

### Cancer profiles and (mortality) trends

**Figure 1:**
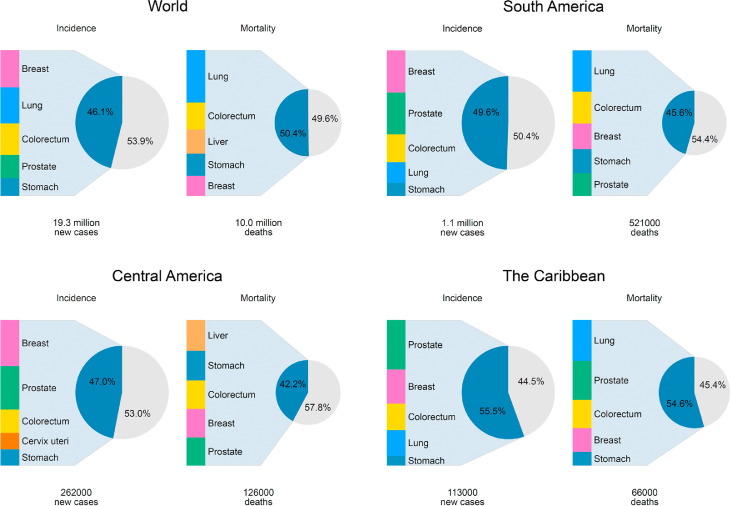
Five most frequent cancers in the world and in LAC by subregions, both sexes combined, incidence and mortality, 2020.

**Figure 2:**
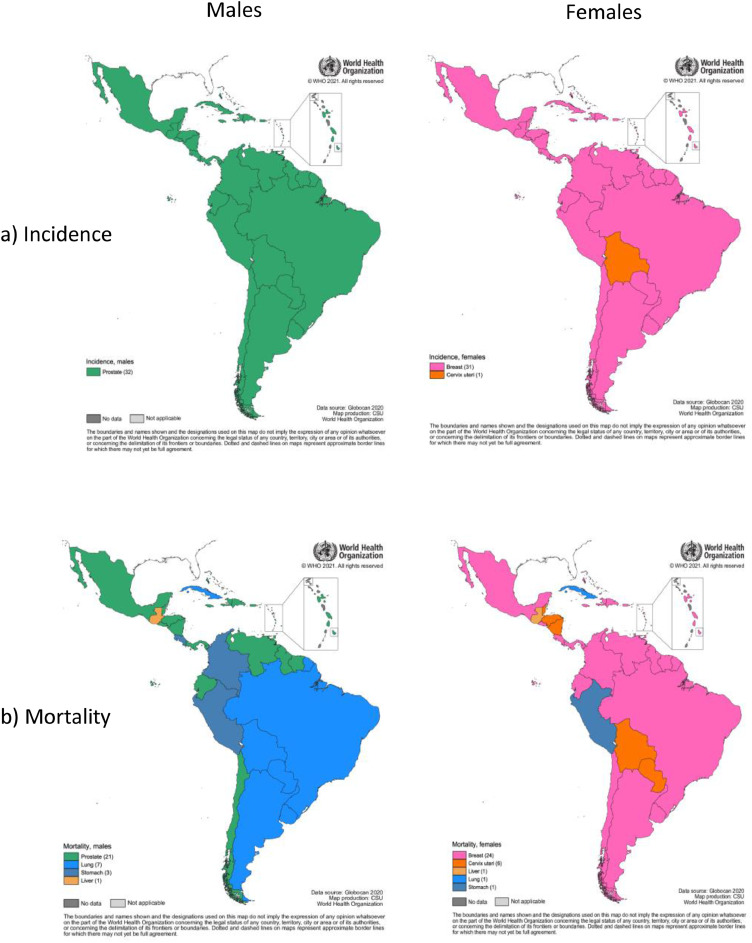
Leading causes of cancer incidence (a) and mortality (b) among males and females by country, LAC 2020.

In South America, Central America, and the Caribbean the five most common cancers respectively constituted 49.6%, 47.4%, and 55.5% of all cancers (Figure 1). Similar to the global cancer profile, the most commonly diagnosed cancers in both South America and the Caribbean, were prostate (15% of all cancers, both sexes), breast (14%), colorectal (9%), lung (7%) and stomach (5%) while lung cancer continued to be the leading cause of cancer deaths for both sexes combined (12%) (Table 2). This pattern was slightly different for Central America, where lung cancer was replaced within the top five leading cancers by cervical cancer (in terms of incidence) and liver cancer (mortality) (Figure 1). Among males, prostate cancer was the most frequently diagnosed cancer in all countries and the leading type of cancer death in 21 countries (Figure 2a, b). Breast cancer was the most frequent incident cancer in women in all countries except Bolivia (Figure 2 a); cervix cancer remained the leading form of cancer mortality among females in six countries (Figure 2b).

**Table 2:** Incidence and mortality of most frequent cancers in LAC region, 2020.

		New cases			Deaths		
ICD	Cancer	Nr	%	ASR (W)*	Nr	%	ASR (W)*
C61	Prostate	214522	14.6	59.2	57415	8.0	14.2
C50	Breast	210100	14.3	51.9	57984	8.1	13.5
C18-21	Colorectum	134943	9.2	16.6	69435	9.7	8.2
C33-34	Lung	97601	6.6	12	86627	12.1	10.5
C16	Stomach	67617	4.6	8.3	53392	7.5	6.4
C73	Thyroid	63368	4.3	8.6	4406	0.6	0.5
C53	Cervixuteri	59439	4.0	14.9	31582	4.4	7.6
C82-86, C96	Non-Hodgkin lymphoma	39886	2.7	5.2	19153	2.7	2.4
C22	Liver	39495	2.7	4.8	37566	5.3	4.6
C91-95	Leukaemia	38256	2.6	5.4	27631	3.9	3.7
C25	Pancreas	37352	2.5	4.5	36030	5.1	4.3
C64-65	Kidney	35990	2.4	4.7	15831	2.2	2.0
C67	Bladder	33840	2.3	4	13100	1.8	1.5
C54	Corpusuteri	33270	2.3	8.2	8718	1.2	2.0
C70-72	Brain,central nervous system	25835	1.8	3.5	22176	3.1	2.9
C56	Ovary	23513	1.6	5.8	15266	2.1	3.6
C15	Oesophagus	19011	1.3	2.4	17799	2.5	2.2
C43	Melanoma of skin	18881	1.3	2.3	5657	0.8	0.7
C00-06	Lip,oral cavity	17888	1.2	2.3	7548	1.1	0.9
C32	Larynx	16140	1.1	2.1	10223	1.4	1.3
C88+C90	Multiple myeloma	15184	1.0	1.9	11289	1.6	1.4
C62	Testis	13653	0.9	3.8	2139	0.3	0.6
C81	Hodgkin lymphoma	10634	0.7	1.5	2835	0.4	0.4
C23	Gallbladder	9990	0.7	1.2	6464	0.9	0.8
C09-10	Oropharynx	8884	0.6	1.2	4915	0.7	0.6
C60	Penis	4988	0.3	1.3	1627	0.2	0.4
C07-08	Salivary glands	4267	0.3	0.54	1303	0.2	0.2
C51	Vulva	3824	0.3	0.84	1382	0.2	0.3
C46	Kaposi sarcoma	2856	0.2	0.37	571	0.1	0.1
C12-13	Hypopharynx	2430	0.2	0.31	1076	0.2	0.1
C11	Nasopharynx	2045	0.1	0.27	1176	0.2	0.2
C52	Vagina	1434	0.1	0.33	494	0.1	0.1
C45	Mesothelioma	1238	0.1	0.16	1082	0.2	0.1
C00-97	All cancers	1470274		186.5	713414		86.5

### Prostate and breast cancer

**Figure 3:**
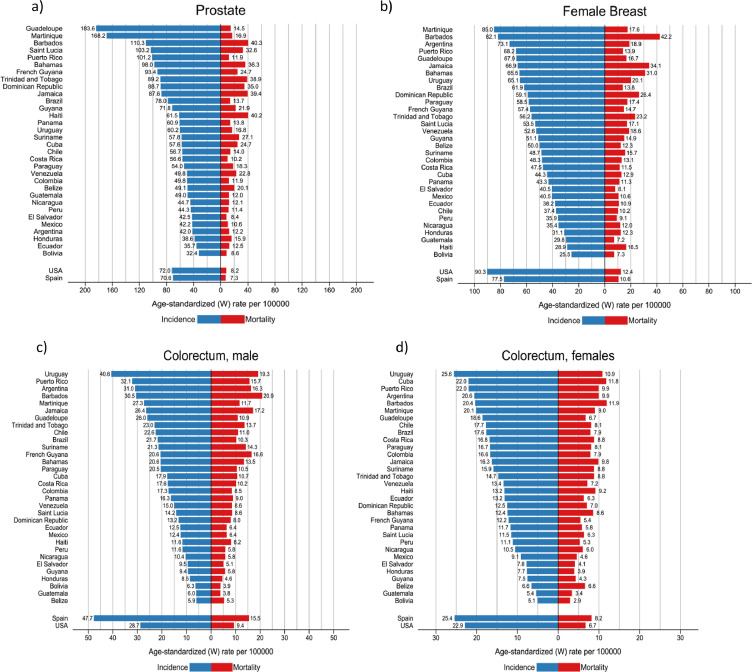

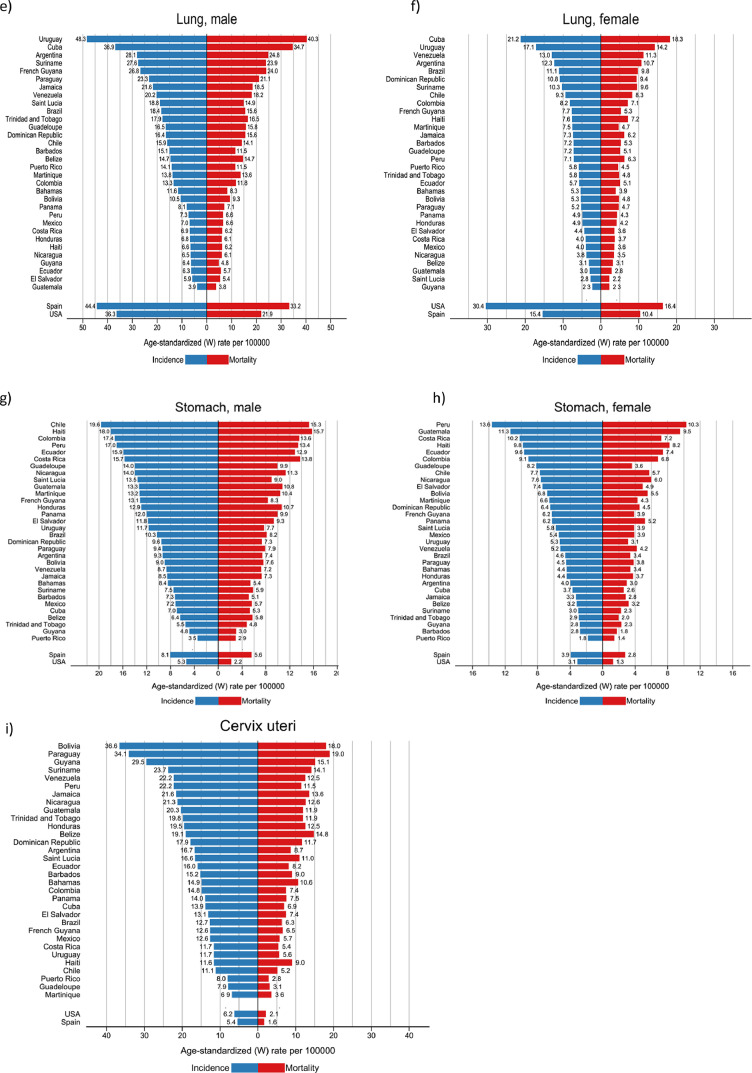
Cancer incidence and mortality rates (ASR per 100,000) for main cancers, by country, LAC, 2020. a) Prostate, b) Female breast c) Colorectal- males d) Colorectal- females e) Lung -males f) Lung -females g) Stomach -males h) Stomach- females i) Cervix uteri. * Scales vary according to cancer site.

**Figure 4:**
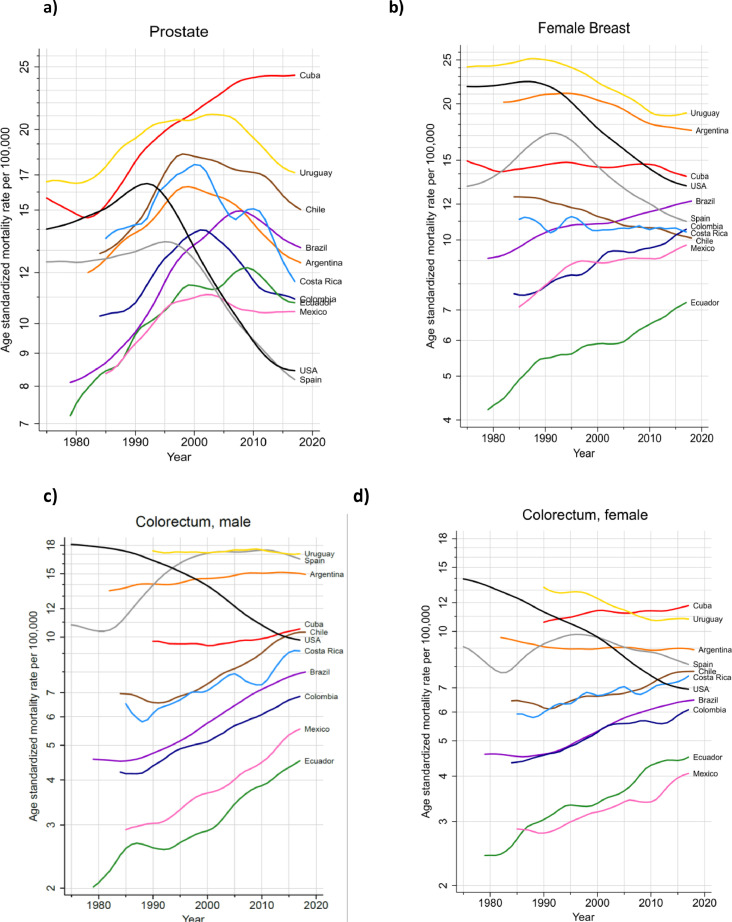

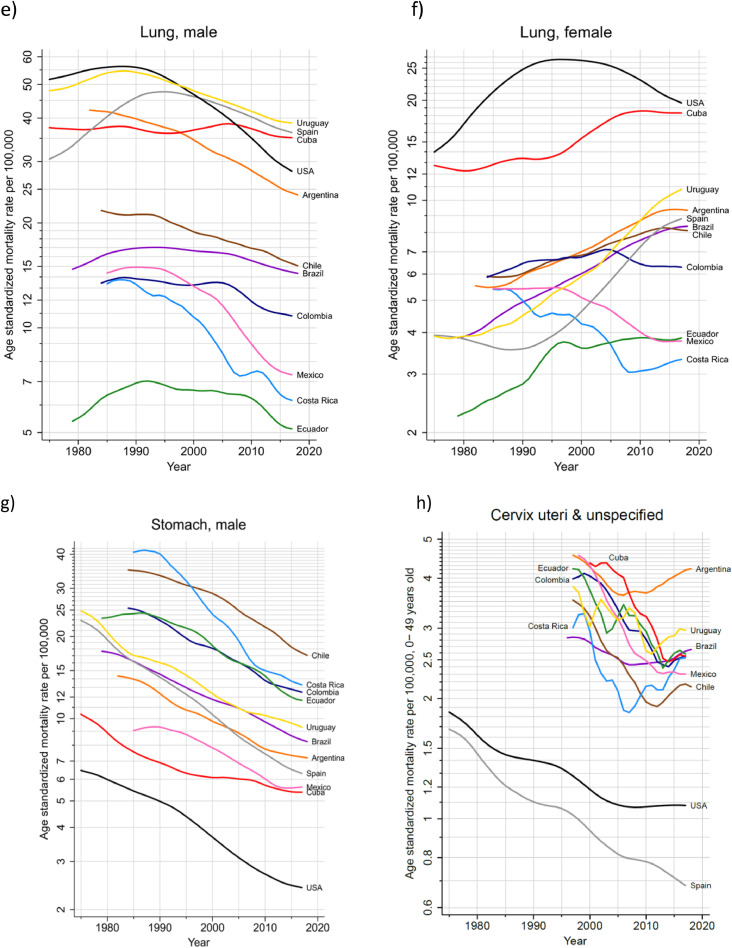
Trends in cancer mortality rates (ASR per 100,000) by country, LAC 2020; a) Prostate b) Breast -female c) Colorectal -males d) Colorectal- females e) Lung -males f) Lung -females g) Stomach -males h) Cervix (C53&C55; in women 0-49 years). * Scales may vary according to cancer site.

The more than 200,000 annual new cases of both prostate and breast cancer represented almost one-third of the cancer incidence burden in the region (Table 2). Prostate cancer incidence rates were highest in the countries of the Caribbean and lowest in South America, ranging from 183 per 100,000 in Guadeloupe to 32 in Bolivia (Figure 3a). Breast cancer incidence rates varied three-fold, with the highest rates (ASR) of 85 estimated in Martinique and lowest of 26 in Bolivia (Figure 3b). The variations in mortality from these two cancers were notably less marked than for incidence, with US mortality rates lower than those observed in most countries in the region. In most countries (Cuba an exception), prostate cancer mortality trends have been declining over the last decade (Figure 4a). For breast cancer, mortality rates have however increased steadily in Brazil, Colombia, Mexico, and Ecuador, while decreasing trends were seen elsewhere (Figure 4b).

### Colorectal cancer

This cancer type ranked third in each of the three subregions (Figure 1), accounting for 140,000 incident cases in both sexes, and one in ten cancer deaths in the region (Table 2). Incidence rates were consistently higher among males than females and in countries of the Southern Cone (Argentina, Chile, Uruguay), and in the Caribbean (Figure 3c and d). Mortality rates increased in most countries in both sexes, except for in Argentina and Uruguay, where recent rates were stable. In contrast, colorectal cancer mortality trends decreased substantially in the U.S. in both sexes, and to some extent in Spain (Figure 4c and d).

### Lung cancer

Rates varied up to 10-fold, with elevated incidence and mortality rates observed in Uruguay, Cuba and Argentina in both males and females, with the lowest rates observed in Central American countries and Guyana (Figure 3e, f). Lung cancer mortality trends diverged markedly by sex; in all countries, except Cuba, mortality rates among males decreased, with greater declines in Mexico and Costa Rica (despite relatively lower rates). For females, mortality trends increased steadily in Uruguay and Brazil, exhibiting similar patterns as their Spanish counterparts. In Cuba, Ecuador and Colombia, trends were stable over the last ten years. In the US, mortality trends decreased both among males and females, in the latter since 2000 (Figure 4e, f).

### Stomach cancer

The highest rates were observed in the Andean countries of South America, with Chile ranking first among males and Peru among females; Haiti and Costa Rica also had elevated rates in both males and females (Figure 3g, h). Mortality trends revealed decreasing rates among males in all countries. Nevertheless, the rate of decline appears to have attenuated in recent years in Argentina, Colombia, Cuba, Ecuador and Mexico (Figure 4e).

### Cervical cancer

Cervical cancer incidence and mortality rates were highest in Bolivia and Paraguay, and lowest in Puerto Rico, Martinique and Guadeloupe; corresponding rates in the US and Spain were uniformly lower (Figure 3i). Cervical cancer mortality trends among young women have been in decline in all studied countries; nevertheless, in the last years increases are observed in several countries, notably in Argentina and Uruguay (Figure 4f)

### Regional burden projections to 2040

**Figure 5:**
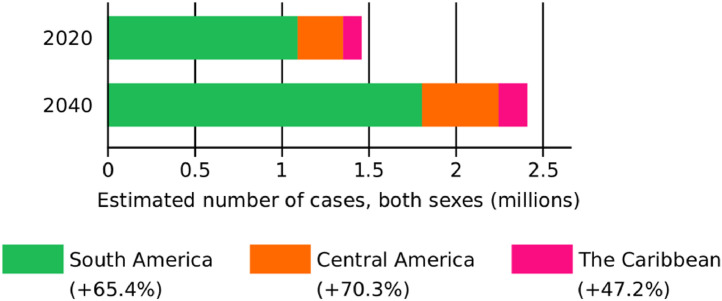
Projections of the cancer incidence burden in LAC by Sub-region, 2020 and 2040.

Figure 5 depicts the expected cancer burden in the region over the next two decades, assuming rates remain unchanged and allowing for the demographic effects of population aging and population growth in LAC. Incidence will rise from 1.5 million new cases to over 2.4 by 2040, implying a close to 66% increase overall – the predicted increase is more marked in Central America (70%) and less so in the Caribbean (47%).

## Discussion

The present profile of the cancer burden as well as the recent trends presented herein reflect a multitude of sociodemographic changes in the LAC region over the last decades, including urbanization and the progressive adoption of more westernized lifestyles at the population level.^17^ While this reflects the leading cancers in all three subregions (prostate, breast and colorectal cancer), infection-related stomach and cervical cancers remain relatively common in the region, particularly in Central America. As a marker of progress in cancer control, there are marked variations in the temporal patterns of cancer-specific mortality, with the broad successes seen in terms of declining trends in stomach, prostate and male lung cancer mortality, tempered by concerns regarding the clear increasing trends in many LAC countries breast and colorectal cancer mortality; for the latter two cancers, the decreases seen in Argentina and Uruguay are the only exceptions.

Within the region, South America exhibited the highest incidence rates for all cancers combined, changing the relative regional position of the 2012 estimates, when the Caribbean ranked first.^5^ This could obey to increase in incidence in South American countries though variations in estimation methods render these comparisons difficult. The higher incidence rates among women observed in a number of countries, contrasts with the observed pattern of higher incidence rates among males which is usually seen high-income settings. This finding may be attributed to several factors, including the remarkable increase in thyroid cancer among females reported by several registries,^18^, ^19^, ^20^ the lower prostate cancer incidence rates in LAC, as well as disproportionately greater health-seeking behaviours and medical consultations among women.^21^ In addition, countries with an elevated female incidence often had a relatively greater percentage of indigenous populations,^22^ a potential contributor to the higher-risk pattern.

The high prostate cancer incidence and mortality rates observed in the Caribbean countries compared to countries in the two other subregions are well-established and confirm previous findings^5^^,^^23^^,^^24^; these patterns, which may underlie more aggressive disease and poorer outcomes have been linked to African ancestry.^25^ While no LAC countries recommend organized prostate cancer screening, little is known on the implementation of opportunistic screening; findings from Brazil however indicate its use may explain the inflated incidence rates in recent years.^26^ The health systems of many Caribbean countries struggle to provide optimal specialized cancer care for their populations,^27^ which may also contribute to the high prostate cancer mortality in the subregion. The favourable declines in prostate cancer mortality observed in most countries of South America coincide with trends in many other settings related to the availability of, and access to, curative treatment.^28^

The relatively high rates of breast cancer incidence and mortality in the Caribbean may also relate to African ancestry and suboptimal cancer care; nevertheless, breast cancer incidence rates also ranked high in Uruguay and Argentina, a pattern that could be associated to factors like European ancestry,^29^ higher educational level^1^ and increased diagnosis as a consequence of the implementation of mass screening programmes. The increasing breast cancer mortality trends in many countries likely reflect a combination of rising incidence combined with a high proportion of advanced stage cancers, particularly among lower educated women and present barriers in access to rapid and effective treatment.^30^, ^31^, ^32^ While it has been claimed that in the region around 40-50% of breast cancers are diagnosed at advanced stages, many of the underlying reports are hospital-, rather than population-based.^33^^,^^34^

Colorectal cancer incidence ranked third in the region, its increasing prominence reflecting the changes in lifestyle factors, particularly diet and physical activity, with high consumption of meat, processed foods and overweight, all of which are established risk factors for colorectal cancer.^35^ The Americas have not only the highest prevalence of adult obesity in the world,^36^ they are also among the world highest meat consumers. This is particularly so for Argentina, Uruguay and some subregions in Brazil that exhibit among the highest colorectal cancer incidence rates in the region.^37^^,^^38^ A high and rising prevalence of the underlying risk determinants is further complicated by limited implementation of early detection policies: only nine LAC countries, for example, have developed guidelines for colorectal cancer early detection with a low adherence reported.^39^ Most countries displayed increasing mortality trends for colorectal cancer that contrasted with the observed declines in the US, Spain and in other high-resource countries.^40^ In Uruguay, the increase in colorectal mortality has been reported among young adults.^38^ Recent predictions of colon cancer mortality to the year 2035 indicate that mortality rates from this cancer will continue to rise in LAC, in contrast to other regions.^41^

Lung cancer continues to be the leading cause of death for both sexes combined in the LAC region, responsible for one in ten cancer deaths. The highest lung cancer incidence rates were seen in those countries associated with a high past and present adult smoking prevalence, namely Uruguay, Cuba and Argentina.^42^ Country- and sex-specific variations in mortality trends largely reflect the different stages of the tobacco epidemic and extent to which tobacco control measures have been adopted. In most countries there is evidence that tax hikes have led to a reduction in smoking prevalence in the last decade^30^; this should result in declining lung cancer mortality rates in future years, as already reported in several countries.^43^^,^^44^

Cervical and stomach cancer continue to be important cancers in most countries and subregions of LAC. While the uniform declines observed in stomach cancer mortality are consistent with the global phenomenon, a recent study in Ecuador indicates stagnation in the stomach cancer mortality (and incidence) rates,^45^ a finding replicated elsewhere and confirmed in our results – the potential increase of cancers of the gastric cardia linked to obesity needs further exploration.^46^ For cervical cancer, the recent increases in mortality among younger women seen in several countries coincides with previous findings^47^ and also warrants investigation. Contrary to our findings, reports by the National Cancer Registry of Uruguay indicate sustained decreasing rates since 1998 in cervical cancer mortality of young women.^48^

Though cervical cancer screening programmes, mostly opportunistic, have been implemented and vaccination strategies adopted in 85% of the countries in the Americas, low coverage remains a major issue in the region,^49^ implying a major scale-up of services will be needed to attain the 4/100,000 incidence threshold set by the WHO Cervical Cancer Elimination Strategy.^50^

The rudimentary predictions provided here imply a large increase in the future cancer burden over the next 20 years. The critical assumption is that rates will remain unchanged 2020-40. A cursory glance of incidence trends^5^ or mortality trends (as was illustrated in Figure 4) in the region implies rates of most cancers are likely to change over the next two decades. We present these however to illustrate the impact of demographic change (population ageing and growth) which will likely lead to an increase in the cancer incidence and mortality burden irrespective of temporal patterns in the corresponding rates. Countries like the U.S. have experienced significant decreases in cancer mortality over the past three decades, attributable to reductions in overall cancer incidence, to advances in early detection, and to improvements in treatment.^51^ A recent IARC modelling study advocated a greater focus on prevention strategies, given policies that tackle tobacco smoking and obesity and the implementation of HPV immunization could avert millions of future cancer diagnoses worldwide.^52^ Although important advances have occurred in tobacco control in the region,^30^ preventive measures such as those targeting physical activity and diet are slow to advance given the enormous challenges to incentivizing the increased levels of exercise and consumption of healthier diets.^53^  With respect to early detection, screening and cancer care, a recent report provides evidence of both progress as well as barriers that persist in the region.^30^ Clearly, the burden predictions forewarn of the need to plan for the provision of oncological care services and the human health force.

As a last point, we would like to underscore that the GLOBOCAN estimates and mortality trends presented here, do not replace the need for continuous recorded data from population-based cancer registries (PBCR). Based on the current availability of PBCR in the region, and particularly of those of high quality, it is clear that major efforts are still needed to attain the overarching aim of the Global Initiative for Cancer Registry Development (GICR, http://gicr.iarc.fr) to inform cancer control through better data. Advocacy efforts are necessary to increase the awareness of governments with regards the sustained value of quality-assured local data. At the same time, improved data capture and reporting needs to be accompanied with IT development systems, while information on critical outcomes, such as stage at diagnosis, are needed to inform necessary advances in early detection. With a paucity of cancer research in LAC relative to the cancer burden,^54^ the full potential of PBCR in building such an evidence base for collaboration needs to be better exploited.

## Contributors

MP was the lead author of this paper, she conceptualized, wrote, edited, reviewed all sections and approved the final version for submission. FB conceptualized, wrote, edited, reviewed all sections and approved the final version for submission. ML produced the figures and tables and approved the final version for submission. MC, EdV, CP and EB wrote, edited, reviewed all sections and approved the final version for submission.

## Data sharing statement

The dataset supporting the conclusions of this article is available at gco.iarc.fr

## Editor's note

*The Lancet Group takes a neutral position with respect to territorial claims in published maps and institutional affiliations.*

## Notes

Where authors are identified as personnel of the International Agency for Research on Cancer / World Health Organization, the authors alone are responsible for the views expressed in this article and they do not necessarily represent the decisions, policy or views of the International Agency for Research on Cancer / World Health Organization.

## Declaration of interests

EdV declared payment or honoraria by AMGEN for presentation on socioeconomic inequalities in Colombia presenting results of projects not related to the industry. All other authors declare that they have no competing interests.
